# Importance of nociplastic pain in patients with rheumatic diseases

**DOI:** 10.1016/j.clinsp.2023.100309

**Published:** 2023-11-10

**Authors:** Antonio Alcántara Montero

**Affiliations:** Centro de Salud Trujillo, Trujillo (Cáceres), Spain

I read the article by Helito CP et al. with great interest. In this article, they evaluate the prevalence and interference of neuropathic pain in the quality of life in patients with knee Osteoarthritis (OA). Neuropathic pain was present in 28.6% of the patients with knee OA who are candidates for arthroplasty. Patients with associated neuropathic pain present a higher level of pain and worse quality of life scores.^1^

In addition to nociceptive and neuropathic pain, the International Association for the Study of Pain (IASP) provided a new term on 14 December 2017: “nociplastic pain”. They define this as “*pain that arises from altered nociception despite no clear evidence of actual or threatened tissue damage causing the activation of peripheral nociceptors or evidence for disease or lesion of the somatosensory system causing the pain*”.^2^ This term serves to contrast with neuropathic pain. Therefore, it describes the pain that occurs in a somatosensory nervous system which functions as normal, contrasting with the poor or abnormal functioning observed in the case of neuropathic pain.^3^ This means the ethiopathogeny of nociplastic pain is complex and highly dependent on psychological factors such as catastrophizing, low perception of self-efficacy, and neuroticism, all of which act as predictors for this type of pain.^4^

The prototypical conditions of nociplastic pain include both generalized (such as Fibromyalgia [FM]) and localized conditions (like chronic temporomandibular pain disorders, primary chronic vesicle pain, irritable bowel syndrome, tension headache, chronic migraine), which are known as conditions of chronic overlapping pain.^3^

It is important to highlight that the term “nociplastic pain” is for clinical use and not for diagnosis. It is certainly not to be used as a synonym for central sensitization of nociception, which is merely a neurophysiological concept.^3^ Central sensitization is taken to increase synaptic activity in the somatosensory neurons of the posterior horn of the spinal cord because of a sustained harmful peripheral stimulus ‒ in other words, tissue, or nerve damage.^5^ Central sensitization provokes a progressive and amplified response which sometimes may not correlate with the painful stimulus intensity. These phenomena manifest clinically through the hypersensitivity to painful stimuli or hyperalgesia and as hypersensitivity to non-painful stimuli or allodynia.^6^

Furthermore, the increase in the nociceptive neuron response capacity in the central or peripheral nervous system, due to central or peripheral sensitization or both, is a well-known physiopathological characteristic of nociceptive and neuropathic pain.^4^ Altered nociception, which is also assumed in the physiopathology of nociplastic pain, is associated with a peripheral and/or central sensitization, which can be clinically differentiated using Quantitative Sensory Testing (QST).^4^ As with neuropathic pain, it is important to evaluate the central sensitization phenomena in rheumatology disorders, as they can be present in approximately 30% of patients with OA.^7^ What's more, its presence implies a different therapeutic approach to that of habitual nociceptive pain. To evaluate the central sensitization, the Central Sensitization Inventory (CSI), which scores between 0 and 100, is most useful. A CSI score of 40 or above indicates the presence of pain with central sensitization.^8^

Patients with rheumatic illness and their healthcare providers often assume all pain to be nociceptive ‒ a direct result of joint inflammation. In reality, several types of pain can contribute to a general feeling of pain (Fig. 1). Thus, nociplastic pain affects a significant subgroup of patients with rheumatic disorders. Given the relative novelty of the term “nociplastic” and that most studies do not evaluate pain sensitization directly, it is not possible to provide prevalence rates for nociplastic pain *per se*. However, several studies have examined the prevalence of FM, which is considered a prototypical condition of generalized nociplastic pain. In patients with rheumatic illness, the prevalence of FM ranges between 10% and 48%. In comparison, the prevalence of FM is approximately 2% to 6% in the general population.^9^

**Fig. 1:**
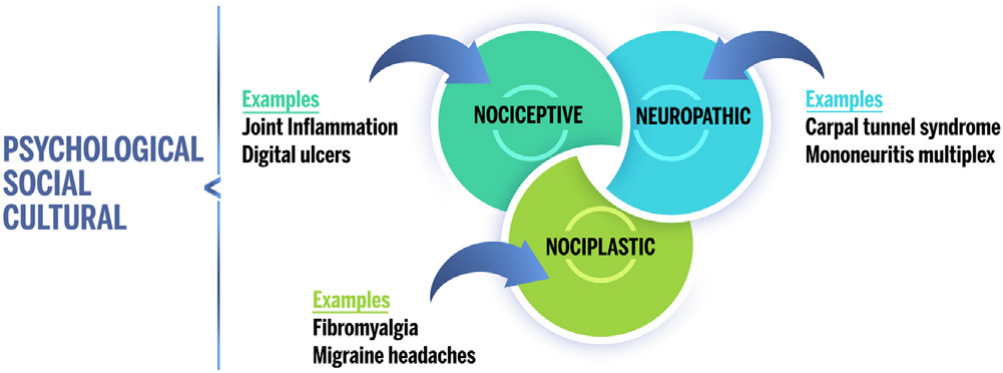
Proposal of chronic pain “descriptors” for the IASP. Pain can be classified into three main categories: nociceptive, neuropathic, and nociplastic. Each individual's overall pain experience may include one or more of these types of pain, which are also modulated by psychological, social, and cultural factors.

Nevertheless, we must not disregard “mixed pain”, which despite having been referenced in literature for almost a decade, has never been formally defined. In fact, the term “nociplastic pain” in IASP terminology excludes patients who clinically present with a substantial overlap of nociceptive and neuropathic symptoms. For these patients, the term “mixed pain” is increasingly recognized and accepted by healthcare professionals. For this reason, a group of international pain specialists from different specialties has recently defined “mixed pain” as: “*a complex overlap of the different known pain types (nociceptive, neuropathic, nociplastic) in any combination, acting simultaneously and/or concurrently to cause pain in the same body area*”.^10^

I hope these reflections help classify the different conditions of chronic pain by evaluating pain sensitization characteristics and the three definitions proposed by the IASP (not forgetting “mixed pain” either) and serve to encourage more in-depth analysis and information to gather new evidence, as well as research which aims to further evaluate the pathogenesis of pain conditions such as rheumatic illnesses.

## Conflicts of interest

The author declare no conflicts of interest.
